# Pregnancy outcomes in women with a hemoglobinopathy trait: a multicenter, retrospective study

**DOI:** 10.1007/s00404-021-06058-y

**Published:** 2021-04-11

**Authors:** Jan Kasparek, Tilo Burkhardt, Irene Hoesli, Gabriela Amstad Bencaiova

**Affiliations:** 1 Department of Obstetrics and Antenatal Care, University Hospital Basel, Spitalstrasse 21, 4031 Basel, Switzerland; 2 Department of Obstetrics and Antenatal Care, University Hospital Zurich, Frauenklinikstrasse 10, 8091 Zurich, Switzerland

**Keywords:** Hemoglobinopathy trait, Pregnancy, Maternal outcomes, Neonatal outcomes

## Abstract

**Purpose:**

To determine the risk of adverse maternal and neonatal outcomes in pregnant women with a hemoglobinopathy trait.

**Materials and methods:**

Retrospective cohort study was conducted to compare adverse maternal and neonatal outcomes between pregnant women with a hemoglobinopathy trait (study group; *n* = 172), and without a hemoglobinopathy trait (control group; *n* = 360). The medical data were extracted from clinical records of pregnant women attending antenatal care and delivering at the University Hospital Basel or University Hospital Zurich between 2015 and 2018.

**Results:**

A total of 172 pregnant women with a hemoglobinopathy trait and 360 controls were recruited. Apart from fetal acidosis, the groups did not differ significantly in any variables of adverse neonatal outcomes. Whereas, among the maternal outcomes the rate of abortion, gestational diabetes mellitus, bacteriuria or urinary tract infection, intrahepatic cholestasis, abnormal placentation and anemia postpartum were significantly increased in women with a hemoglobinopathy trait.

**Conclusion:**

In our study, a hemoglobinopathy trait increased the risk of adverse maternal outcomes but did not increase adverse neonatal outcomes.

## Introduction

Anemia is the most common disease in pregnancy and postpartum. The most common causes of anemia among pregnant women are iron deficiency and inherited hemoglobin disorders. The World health organization (WHO) estimates that overall at least 5.2% of the world population carry a significant variant of a hemoglobin disorder; among pregnant women the prevalence is even higher with over 7% [1]. Inherited hemoglobin disorders were originally characteristics of the tropics and subtropics; however, due to migration they are now common worldwide and thus presenting an increasing global health burden.

Over a number of years, several studies on the effect of a hemoglobinopathy trait on pregnancy have been published. Although commonly known that anemia in pregnancy is a significant risk factor of adverse maternal and fetal outcomes, previous studies on the effect of a hemoglobinopathy trait on pregnancy provide controversial results and inconsistent conclusions. Some studies suggest an increased risk of adverse pregnancy outcomes [2–5], whereas others cannot confirm this [6–9]. There are studies showing an increased maternal risk of prevalence of bacteriuria [5, 9–11], gestational diabetes mellitus [5, 12, 13], anemia [2, 11], pre-eclampsia [14, 15] thromboembolism [16], spontaneous abortion [17], and an increased rate of caesarean section [18, 19]. For the fetus, increased risk of oligohydramnion [18, 19], low birth weight [17, 20, 21], small for gestational age babies [21] and intrauterine growth restriction [6, 9, 22], have been described in some studies. Other studies could not verify these increased risks for the fetus among women with a hemoglobinopathy trait [2, 5, 6, 9, 15].

It is unclear whether the hemoglobinopathy trait increases the rates of adverse pregnancy outcomes. To examine this association, we conducted a study to compare the adverse pregnancy outcomes in pregnant women with and without a hemoglobinopathy trait. To our knowledge, the association between the hemoglobinopathy trait and intrahepatic cholestasis, abnormal placentation and puerperal infection has been investigated for the first time.

## Materials and methods

This multicenter, retrospective cohort study was conducted at the University Hospital Basel and University Hospital Zurich, Switzerland, Department of Obstetrics and Antenatal Care between 2015 and 2018. The medical data were extracted from the patient’s clinical records. The study received ethical approval from the Swiss local ethics committee in Basel and Zurich (ID 2018–00101) and was registered under http://www.ClinicalTrials.gov (NCT03837119) on 11 February 2019.

A total of 532 pregnant women attending antenatal care and delivering at the University Hospital Basel or University Hospital Zurich were included in this study. Pregnant women were recruited from outpatients’ department at the University Hospital Basel and anemia consultation at the University Hospital Zurich. Family origin questionnaires were used to screen pregnant women for the risk of hemoglobinopathies in the first trimester at the University Hospital Basel. The Family origin questionnaire was adopted from the National Health Screening: Sickle Cell and Thalassemia Screening Programme in England [23]. Based on the questionnaire, pregnant women were divided into two groups: women with a high risk and women with a low risk of hemoglobinopathies. In women with a high risk, red blood cell indices, iron status and high-performance liquid chromatography (HPLC) were conducted. At the University Hospital Zurich, women with persistent anemia, with a poor response to iron therapy or low MCH were examined by HPLC. For women identified as carriers, their partner was also tested for hemoglobinopathy, irrespective of family origin. In cases where alpha thalassemia was suspected based on hematological parameters (MCH < 27 pg regardless of iron status), a molecular analysis was performed. If both were carriers of hemoglobinopathies, genetic counselling was recommended and an antenatal genetic testing via choriovillous sampling or amniocentesis was discussed with the patient.

Adverse maternal and neonatal outcomes were examined among pregnant women with and without a hemoglobinopathy trait. Inclusion criteria for the study group were healthy women with a hemoglobinopathy trait, without comorbidities and age ≥ 18 years (study group; *n* = 172). The control group included women without a hemoglobinopathy trait and comorbidities. The matching criteria were maternal age, parity and gestational age at delivery. The pregnant women were randomly selected using a ratio of 1:2 (study group: control group; *n* = 360).

The primary outcome was the prevalence of adverse neonatal outcomes. The secondary outcome was the prevalence of adverse maternal outcomes. Gestational diabetes mellitus (GDM) was defined using a universal one-step screening strategy and according to the results of an oral 75 g glucose tolerance test (oGTT). The 75 g-oGTT was discontinued in the case of fasting plasma glucose < 4.4 mmol/l due to the unlikelihood of gestational diabetes (sensitivity 95%) [24–26]. For the diagnosis of an intrahepatic cholestasis of pregnancy, a cut-off value of total serum bile acid 10 µmol/l was used [27]. Asymptomatic bacteriuria was defined by a significant growth of pathogens in a urine culture that is greater than 10^5^ bacteria/ml, but without the patient showing symptoms of a urinary tract infection [28, 29].

The following maternal secondary objectives were examined: rate of abortions, pre-eclampsia (defined according to the “Report of the American College of Obstetricians and Gynecologists’ Task Force on Hypertension in pregnancy”), pregnancy-induced hypertension [30], mode of delivery, abnormal placentation, placental abruption, peripartum hemorrhage, postpartum anemia, and puerperal infection or sepsis. Abnormal placentation included placenta praevia and placenta accreta [31]. Placenta accreta was defined by the presence of the following: (1) prenatal diagnosis of abnormal placental invasion by ultrasound imaging with clinical or histopathological confirmation or (2) difficult manual, piecemeal removal of the placenta or “prolonged third stage of labor” if no evidence of placental separation was noticed more than 30 or 60 min after delivery, despite active management of the third stage of labor. Peripartum hemorrhage was defined by blood loss of ≥ 500 ml following vaginal delivery or ≥ 1000 ml following caesarean section [32]. According to current guidelines recommended by the Centre for Disease Control (CDC, USA), anemia in pregnancy was defined by a hemoglobin (Hb) of less than 110 g/l [33].

The following neonatal outcomes were investigated: gestational age at birth, birth weight, preterm delivery < 37 weeks of gestation, preterm premature rupture of membranes (PPROM < 37 weeks of gestation), macrosomia defined as birth weight above 97th percentile or birth weight above 4300 g [34], intrauterine growth restriction (IUGR) defined as birth weight below 3rd percentile [35], low birth weight (LBW) with a birth weight below 2500 g, Apgar score < 5 at 5, fetal acidosis defining by arterial pH < 7.15, stillbirth and neonatal death defined according to UNICEF and WHO, and admissions to the neonatal unit care (NICU). The measurement of arterial and venous pH was performed in all newborns. The gestational age was assessed according to the last menstruation date or adjusted through a first trimester ultrasound if the discrepancy was more than ± 5 days.

Pregnant women in Switzerland are supplemented with a multivitamin. The multivitamin, specifically for pregnant women, contains a divalent iron between 27 and 60 mg per pill. Women with iron deficiency were treated with oral iron supplements. Intravenous iron therapy was administered if Hb < 90 g/l and a concomitant iron deficiency, poor compliance or intolerance to oral iron therapy, or a poor response to oral iron therapy. If Hb < 80 g/l, recombinant human erythropoietin was administered in addition to intravenous iron. No woman was treated with blood transfusion during pregnancy.

### Hematological assessment

All blood measurements (blood count, CRP, ferritin) were conducted at the University Hospital of Basel and Zurich, Department of Laboratory Medicine.

Hemoglobin, red blood cell count (RBC), hematocrit (HCT), mean corpuscular volume (MCV), mean corpuscular hemoglobin (MCH), microcytic red blood cells (MRC), hypochromic red blood cells (HRC) and red blood distribution width (RDW) were measured using a hematology analyser. The mean corpuscular hemoglobin was automatically calculated from Hb and RBC counts.

Serum ferritin was assessed by chemiluminescence immunoassay and C-reactive protein (CRP) was assessed by immunoturbidimetry.

### Statistical analysis

Statistical analysis was conducted using STATA 12.0 (Stata Corporation College Station, TX). Continuous variables were compared using Student’s *t* test for different sample sizes. Nominal and categorical variables were compared using the *χ*^2^ test. The level of statistical significance was set at *p* < 0.05. Odds ratios (ORs) with 95% confidence intervals were calculated for infant outcome variables and delivery mode.

## Results

The demographic data of the groups are shown in Table 1. Apart from gravidity and BMI, baseline characteristics of the two groups were not significantly different. Increased gravidity in the study group could be due to an attributed to a higher spontaneous abortion rate in women with a hemoglobinopathy trait. In the study groups, four women with twin pregnancy were included (*n* = 176). There were 22 women with BMI ≥ 30 kg/m^2^ in study group, including 3 women with BMI ≥ 40 kg/m^2^. In the control group, there were 17 women with BMI ≥ 30 kg/m^2^, including 3 women with BMI ≥ 40 kg/m^2^.

**Table 1:** Characteristics of the study and control group

	Study group (*n* = 172)	Control group (*n* = 360)	*p* value
Maternal age (years)	31.5 ± 5.8 (18–49)	31 ± 3.2 (26–36)	0.202
Gravidity	2.6 ± 1.8 (1–13)	2.0 ± 0.9 (1–4)	< 0.001
Parity	2.0 ± 1.3 (1–11)	2.0 ± 0.7 (1–3)	1.000
BMI (kg/m^2^)	24.4 ± 5 (16–42.5)	23 ± 5 (16–63)	0.003
Gestational age at delivery (weeks)	38.1 ± 3 (26–42)	38.3 ± 3 (24–41)	0.472
< 37 (%)	22/172 (12.8)	52/360 (14.5)	0.688
37–42 (%)	150/172 (87.2)	308/360 (85.5)	0.688

Data expressed as mean ± sd (range) or *n* (%)

Out of the 172 women with a hemoglobinopathy trait, there were 24 women with sickle cell trait, 40 with alpha thalassemia, 84 with heterozygous beta thalassemia, 3 with heterozygous delta thalassemia, 12 with hemoglobinopathy E, 1 with hemoglobinopathy D, 2 with hemoglobinopathy C and 6 with compound hemoglobinopathy. Of 172 women with a hemoglobinopathy trait, 87 were treated at the University Hospital Basel and 85 at the University Hospital Zurich.

**Fig. 1:**
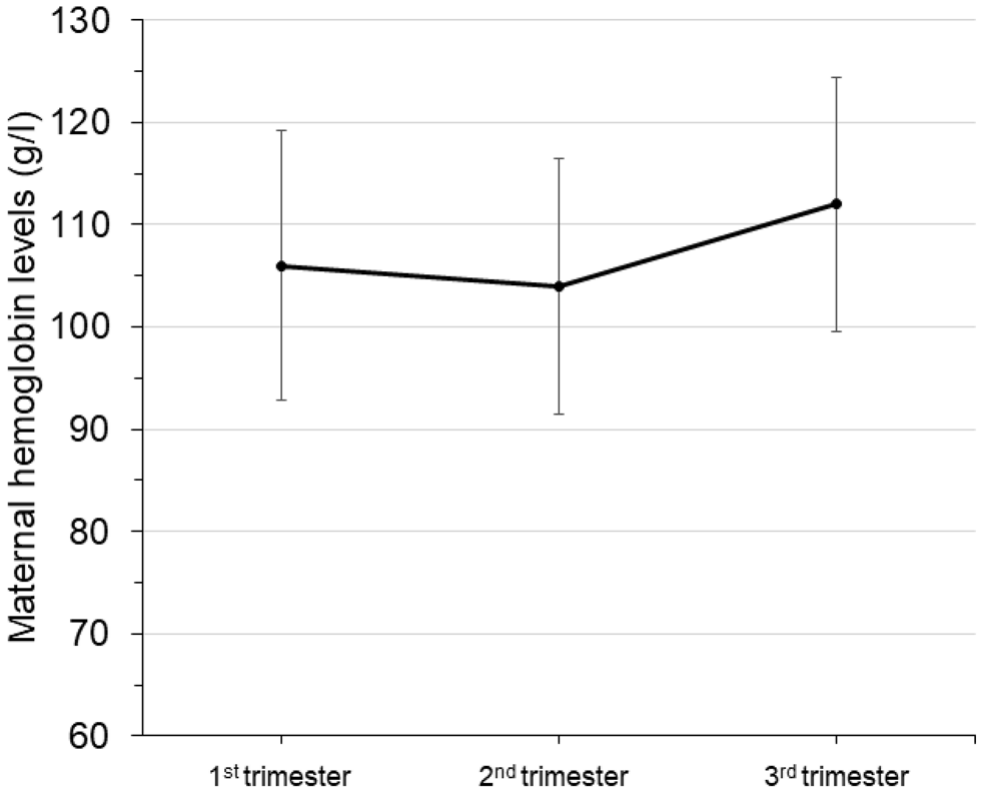
The course of hemoglobin during pregnancy in women with a hemoglobinopathy trait

The mean of hemoglobin in the study group in the first trimester was 106 ± 13 g/l (72–144 g/l) and the median of ferritin 41 µg/l (4–623 µg/l). The mean of hemoglobin, serum ferritin and CRP of the study group are shown in Table 2; the origin of women with a hemoglobinopathy trait in Table 3. Anemia was identified in 59.1% of women with a hemoglobinopathy trait in the first trimester (94/159) and iron deficiency in 39.6% (57/144) (Table 2). The course of hemoglobin levels during pregnancy in women with hemoglobinopathy is shown in Fig. 1.

**Table 2:** Hematological data and serum iron status of the study group in the first trimester

	Study group (*n* = 172)
Hb (g/l)	106 ± 13.2 (72–144)
RBC (× 10^6^/µl)	4.59 ± 0.6 (2.65–6.21)
Anemia (%)	94/159 (59.1)
HCT (%)	32.5 ± 3.8 (23–42)
MCV (fl)	71.3 ± 9.0 (55.9–104)
MCH (pg)	23.7 ± 3.8 (16.3–35.5)
MRC (%)	5.1 (0–72.3)
HRC (%)	23.5 (0–69.5)
RDW (%)	15.7 ± 1.9 (12.2–20.5)
Ferritin (µg/l)	41 (4–623)
Iron deficiency (%)	57/144 (39.6)
CRP (mg/l)	4.2 (0.4–74)

Data expressed as mean ± sd (range) or *n* (%)

**Table 3:** The origin of women with a hemoglobinopathy trait

Origin	*N*
Mediterranean	55
Italy	29
Spain	4
Greece/Cyprus	4
Portugal	3
Other	15
Middle East and North Africa	22
Iran/Syria	3
Turkey	11
North Africa	8
South Asia	31
Bangladesh	5
Indian	15
Pakistan	3
Sri Lanka	8
South East Asia	20
Indonesia/Vietnam/ Philippine	6
Thailand	11
China	3
Africa/Caribbean	38
Other	6

There were no differences regarding delivery mode and in rate of caesarean sections (49.4% vs. 43.6%; *p* = 0.227, RR 1.26, 95% CI 0.86–1.85) (Table 4). Excluding fetal acidosis (14.9 vs. 6.0%; *p* = 0.012) the groups did not differ significantly in any other variable of adverse neonatal outcomes (Table 5). Only one neonate in the study group showed a pH ≤ 7.00.

**Table 4:** Delivery mode

Outcome *n* (%)	Study group (*n* = 172)	Control group (*n* = 360)	RR (95% CI)	*p* value
Vaginal delivery	87/172 (50.6)	203/360 (56.3)	0.79 (0.54–1.16)	0.227
Non-operative vaginal delivery	74/172 (43.0)	163/360 (45.3)	0.91 (0.62–1.34)	0.642
Operative vaginal delivery	13/172 (7.6)	40/360 (11.1)	0.65 (0.31–1.29)	0.219
Caesarean section	85/172 (49.4)	157/360 (43.6)	1.26 (0.86–1.85	0.227
Elective caesarean section	47/172 (27.3)	99/360 (27.5)	0.99 (0.64–1.52)	1.000
Non-elective caesarean section	38/172 (22.1)	58/360 (16.1)	1.48 (0.91–2.38	0.117

**Table 5:** Neonatal outcomes

	Study group (*n* = 176)	Control group (*n* = 360)	*p* value
Birth weight (g)	3119 ± 620 (880–4685)	3135 ± 694 (420–4840)	0.797
PPROM	3/87 (3.44)	14/360 (3.9)	0.186
Low birth weight	22/176 (12.5)	41/360 (11.4)	0.668
IUGR	9/176 (5.1)	14/360 (3.9)	0.498
Macrosomia	4/176 (2.3)	13/360 (3.6)	0.600
Apgar score < 5 at 5	4/176 (2.3)	7/360 (2.0)	0.753
Fetal acidosis	13/87 (14.9)	20/335 (6.0)	0.012
NICU admission	9/87 (10.4)	45/360 (12.5)	0.715
Early neonatal death	1/176 (0.6)	2/360 (0.6)	1.000
Still birth	0/176	1/360 (0.3)	1.000

Data expressed as mean ± sd (range) or *n* (%)

Contrary to an adverse neonatal outcome, we have found a significant difference in adverse maternal outcomes (Table 6). However, due to incomplete data, only women treated at the University Hospital Basel were included for this analysis (*n* = 87). There was a significant difference in the occurrence of previous spontaneous abortion (31.4 vs 21.4%; *p* = 0.014, RR 1.68, 95% CI 1.09–2.58), gestational diabetes mellitus (27.6 vs 11.1%; *p* < 0.001, RR 3.05, 95% CI 1.63–5.59), bacteriuria and urinary tract infection (21.7 vs 2.2%; *p* < 0.001, RR 12.18, 95% CI 4.76–33.51), intrahepatic cholestasis (11.5 vs 2.2%; *p* < 0.001, RR 5.71, 95% CI 1.94–17.16), abnormal placentation (11.5 vs 8.1%; *p* = 0.009, RR 4.41, 95% CI 1.28–15.13) and postpartum anemia (80.2 vs 45.3%; *p* < 0.001, RR 4.90, 95% CI 2.71–9.24), with a significant difference in hemoglobin postpartum (Hb postpartum in the study group was 96 ± 15 g/l (47–128) compared to 110 ± 13 g/l (71–141) in the control group; *p* < 0.001). There was no difference in serum ferritin between women with and without GDM in the study group (median of serum ferritin in women with GDM was 57 µg/l [7–306] vs. in women without GDM 60 µg/l [7–623]).

**Table 6:** Maternal complications

	Study group (*n* = 87)	Control group (*n* = 360)	RR (95% CI)	*p* value
Abortion	54/172 (31.4)	77/360 (21.4)	1.68 (1.09–2.58)	0.014
Gestational diabetes	24/87 (27.6)	40/360 (11.1)	3.05(1.63–5.59)	< 0.001
Bacteriuria or urinary tract infection	18/83 (21.7)	8/360 (2.2)	12.18 (4.76–33.51)	< 0.001
Intrahepatic cholestasis	10/87 (11.5)	8/360 (2.2)	5.71 (1.94–17.16)	< 0.001
Hypertensive disorders	1/87 (1.2)	4/360 (1.1)	1.03 (0.02–10.6)	1.000
Pre-eclampsia/eclampsia	4/87 (4.6)	15/360 (4.2)	1.10 0.26–3.6)	0.772
Abnormal placentation	7/87 (8.1)	7/360 (2.0)	4.41 (1.28–15.13)	0.009
Premature placental abruption	0/87 (0)	5/360 (1.4)	n. a	0.588
Peripartal hemorrhage	19/172 (11.5)	29/360 (8.1)	1.42 (0.73–2.71)	0.262
Anemia postpartum	69/86 (80.2)	154/340 (45.3)	4.90 (2.71–9.24)	< 0.001
Infection postpartum	2/86 (2.3)	3/360 (0.8)	2.83 (0.23–25.1)	0.248

Data expressed as *n* (%)

All kinds of the hemoglobinopathy trait were consistently affected by the adverse maternal outcomes; in this regard, there was no preferential kind of hemoglobinopathy. Subgroup analysis has not shown any differences between different types of the hemoglobinopathy trait.

## Discussion

This study suggests that a hemoglobinopathy trait might have an effect on some maternal complications, but does not increase adverse neonatal outcomes.

Most baseline characteristics of the two groups were comparable, but gravidity and BMI were significantly higher in the study group. Concerning the significant differences in gravidity and BMI between the groups, a possible bias in the interpretation of the results must be considered, as these characteristics represent specific pregnancy outcome risks themselves. However, increased gravidity in the study group is mainly caused by a higher abortion rate in women with a hemoglobinopathy trait, likewise corresponding with previous findings by Charoenboon et al. [17]. On the other hand, although higher BMI has been shown in the study group, the mean BMI of both groups was in the normal range. Based on the magnitude of adverse pregnancy outcomes increasing with higher BMI value [36, 37], a normal mean BMI does not explain the significant difference in adverse maternal outcomes such as GDM, cholestasis or urinary tract infection between both groups.

Apart from fetal acidosis, the groups did not differ significantly in any examined variable of adverse neonatal outcomes. The increase of fetal acidosis could be caused by complications in women with a hemoglobinopathy trait and thereby associated placental insufficiency.

There are many studies with controversial results on the risk of gestational diabetes among women with a hemoglobinopathy trait. Findings in our study indicated that the hemoglobinopathy trait may have an effect on developing gestational diabetes as already suggested in previous studies [5, 12, 13]; however, our study did not have enough power to explicitly express this. Therefore, further studies with larger sample sizes are needed to answer this issue. The controversial findings in respect to gestational diabetes in pregnancy may also be explained by a lack of the standardized screening tests and diagnostic criteria for gestational diabetes in pregnancy worldwide.

Our results concerning asymptomatic bacteriuria or urinary tract infection and anemia in pregnancy and postpartum correspond to the systematic review by Jans et al. [11], and to previous studies reporting a generally higher risk of these complications [2, 5, 9, 10].

To our knowledge, this is the first study examining the prevalence of intrahepatic cholestasis and abnormal placentation in women with a hemoglobinopathy trait. Intrahepatic cholestasis and abnormal placentation were occurred with a higher prevalence in the study group, compared to the control group. However, the number of cases was low with a wide range of 95% CI of the relative risk. The reason for intrahepatic cholestasis increase is unclear, and such an association should be confirmed by further prospective studies with simultaneous determination of liver enzymes.

The prevalence of maternal outcomes including hypertensive disorders, pre-eclampsia/eclampsia, premature placental abruption, peripartum hemorrhage and infection postpartum, were not increased among pregnancies with a hemoglobinopathy trait.

Limitations of this study included a small sample size to gain power in differentiating rates of maternal adverse outcomes, a retrospective approach in which several records contained missing, or not perfectly reliable data, as well as inhomogeneity of the study group. However, the study group is heterogeneous, we do not expect differences between different hemoglobinopathies, since all women were heterozygous carriers for hemoglobinopathy only and different hemoglobinopathies show the same effect on the body and finally pregnancy. On the other hand, to the best of our knowledge, this is the first study examining adverse pregnancy outcomes such as intrahepatic cholestasis, abnormal placentation and infection postpartum between pregnancies, affected and not affected, by a hemoglobinopathy trait.

In conclusion, this study provided evidence that the hemoglobinopathy trait do not increase the rate of adverse neonatal outcomes, whereas the prevalence of maternal complications such as GDM, abortion, urinary tract infection, intrahepatic cholestasis, anemia postpartum and abnormal placentation was significantly increased. Our results could initiate the planning and conducting of prospective powered studies to verify the potential association between the hemoglobinopathy trait and adverse maternal outcomes.

## Data Availability

Data are available on reasonable request.
